# Analysis of Relative Concentration of Ethanol and Other Odorous Compounds (OCs) Emitted from the Working Surface at a Landfill in China

**DOI:** 10.1371/journal.pone.0119305

**Published:** 2015-03-13

**Authors:** Dong Li, Wenjing Lu, Yanjun Liu, Hanwen Guo, Sai Xu, Zhongyuan Ming, Hongtao Wang

**Affiliations:** School of Environment, Tsinghua University, Beijing, 100084, China; The Ohio State University, UNITED STATES

## Abstract

Estimating odor emissions from landfill sites is a complicated task because of the various chemical and biological species that exist in landfill gases. In this study, the relative concentration of ethanol and other odorous compounds emitted from the working surface at a landfill in China was analyzed. Gas sampling was conducted at the landfill on a number of selected days from March 2012 to March 2014, which represented different periods throughout the two years. A total of 41, 59, 66, 54, 63, 54, 41, and 42 species of odorous compounds were identified and quantified in eight sampling activities, respectively; a number of 86 species of odorous compounds were identified and quantified all together in the study. The measured odorous compounds were classified into six different categories (Oxygenated compounds, Halogenated compounds, Terpenes, Sulfur compounds, Aromatics, and Hydrocarbons). The total average concentrations of the oxygenated compounds, sulfur compounds, aromatics, halogenated compounds, hydrocarbons, and terpenes were 2.450 mg/m^3^, 0.246 mg/m^3^, 0.203 mg/m^3^, 0.319 mg/m^3^, 0.530 mg/m^3^, and 0.217 mg/m^3^, respectively. The relative concentrations of 59 odorous compounds with respect to the concentration of ethyl alcohol (1000 ppm) were determined. The dominant contaminants that cause odor pollution around the landfill are ethyl sulfide, methyl mercaptan, acetaldehyde, and hydrogen sulfide; dimethyl disulfide and dimethyl sulfide also contribute to the pollution to a certain degree.

## Introduction

Waste disposal processes produce both chemical and biological emissions in the environment and may expose the residents of nearby communities to certain health and olfactory problems. Chemical and biological emissions from waste disposal processes are perceived as odor pollution; they occur occasionally, repeatedly, or continuously and vary strongly in intensity [1]. Odor pollution has become increasingly serious, particularly in developing countries, such as China, where municipal solid wastes are collected and dumped together; this situation has in turn rendered the siting and operation of landfills difficult. Odorous compounds that are released from landfill sites into the atmosphere potentially cause olfactory nuisances as well as environmental risks within the surrounding communities. Landfill sites are often located in complex topographies for conveniences mainly related to waste disposal and environmental masking [2]. An integrated risk assessment study has been performed in an area within 5 km from a landfill in south Italy that accepts non-hazardous waste [3]. And a health risk assessment methodology was employed to evaluate the potential adverse health effects of the individual compounds according to their carcinogenicities in Turkey [4]. In addition, chemical and biological emissions from landfills can contribute to global warming, stratospheric ozone depletion, tropospheric ozone formation [4], and particle pollution [5].

A large number of studies are investigating the generation of odorous compounds from landfills in both field and laboratory settings; these compounds are often referred to as volatile organic compounds (VOCs) [6], [7], non-methane organic compounds (NMOCs) [8–10], non-methane hydrocarbon (NMHC) [11], or odorous gas (OG) [12]. Most studies mainly focused on the composition and concentration of the odorous compounds. A total of 68 volatile compounds were identified in a landfill in Hangzhou, China [13]. The trace VOCs in landfill gas at seven UK waste disposal facilities was examined and over 140 compounds were identified and quantified [14]. The composition of two different areas in a French landfill site was investigated and 37 NMOCs were quantified [8]. Except for the complicated composition in the landfill gas, there are some spatial variations of these odorous compounds. It is found that the concentration of the odorants varied and strongly depended on the sampling site and the concentrations were influenced by landfill activities, such as the failure of the landfill gas collection system, heavy truck traffic, machinery operations, and compacting fresh waste [12], [15–17]. Besides, there are also temporal variations of trace compounds emitted from landfills. The highest concentrations of odorous compounds were found in summer, probably because of accelerated volatilization under higher ambient temperatures [18], [19].

Estimating odor emissions from landfill sites is a complicated task because of the various chemical and biological species that exist in landfill gases. Gallego et al. [20] used a self-designed cylindrical air flux chamber to determine the emission rates of 60 different VOCs in a landfill located in Spain. Efthimios et al. [21] used methane as an odor index to estimate odors around landfill sites based on the ratio of methane to odorous chemical species. The Landfill Gas Emissions Model (LandGEM) developed by the U. S. EPA also uses methane as an index to estimate the trace compounds emitted from landfills in the USA and lists the relative concentrations of the trace compounds. LandGEM [22] is an automated estimation tool with a Microsoft Excel interface that could be used to estimate the emission rates for total landfill gas, methane, carbon dioxide, NMOCs, and individual air pollutants, including 46 species of trace compounds, from municipal solid waste landfills. LandGEM is based on a first-order decomposition rate equation for quantifying emissions from the decomposition of landfilled waste in MSW landfills. The software provides a relatively simple approach to estimating landfill gas emissions. Model defaults are based on empirical data from U.S. landfills. Field test data can also be used in place of model defaults when available. Landfill gas contains low concentrations of trace pollutants from the leaching and decomposition of wastes, and LandGEM can estimate the emissions of such trace pollutants. The emission of trace pollutants is based on the concentrations of trace compounds in landfill gases. The model contains default concentrations for specific trace pollutant compounds. The list of trace pollutants expected to be emitted with 46 landfill gases and the concentrations of these trace air pollutants were obtained from the test data in AP-42 [23].

As mentioned above and in a large number of other studies, considerable research has been conducted on odorous compounds emitted from landfills, and their adverse effects on the environment and human health, such as olfactory nuisance and carcinogenicity. Most of these studies focus on the composition and concentration of odorous compounds. However, only few results have been reported on the relative concentration of odorous compounds. Although LandGEM gives a list of relative concentration of 46 species of trace compounds, the applicability of such list to landfill working surface conditions in developing countries is still unclear due to the following reasons: (1) LandGEM took into account all emissions from landfills, while the current study focuses mainly on the emissions from the working surface where garbage is perishable and chiefly contributes to odor pollution in the early degradation phase. (2) The landfills that investigated by LandGEM had been in the methanogenic phase and therefore LandGEM used methane as an index, however the working surface is in the early degradation phase, and it does not generate methane while produces abundant odorous compounds. (3) The components of wastes produced in China and other developing Asian countries, which contain higher perishables, significantly differ from those produced in developed countries which contain higher waste paper etc. Thus, using methane as an index to estimate the emission of odorous compounds from the working surface does not fit well our specific situation. It is relevant to find a new index instead of methane to characterize the relative concentration of odorous compounds emitted from the working surface of landfills in developing countries. According to the experimental results from the field and laboratory, we found that the concentration of ethyl alcohol released from the working surface in landfills of China is very high. This high concentration facilitates easy checkout and test; therefore ethyl alcohol could be employed as a new index to estimate the emission of other compounds. This concentration of ethyl alcohol also facilitates estimating the approximate concentration of other odorous compounds, depending on the concentration of ethyl alcohol. The main objective of the current study is to find the correlations among the odorous compounds using statistical tools. This study investigates the odorous compounds emitted from the working surface at a landfill in Beijing, China. The composition and the distribution pattern of the concentration of the various detected odorous compounds are determined, and the relative concentrations of ethyl alcohol and other 59 species of odorous compounds are identified. These results would cast a new light on estimating the contributions of trace compounds to odor pollution.

## Materials and Methods

### Landfill site description

The sampling campaign was conducted in the Asuwei landfill site, which is the largest sanitary landfill in Beijing. The Asuwei landfill site belongs to Beijing Environmental Sanitation Engineering Group Co., Ltd, which granted the permission to conduct the study on this site. The landfill site was constructed and began its operations in 1994; it has an area of about 60.4 ha and a designed filling capacity of 1.19×10^7^ m^3^. The landfill receives approximately 2300 tons of municipal solid waste per day; 10.6 million m^3^ (about 90%) of the landfill’s total area had been filled by the end of 2013. This landfill is expected to be closed in at most three years given the current waste deposition pressure. The landfill mainly handles the municipal solid waste coming from several districts of Beijing. The compacted waste is covered with a high-density polyethylene membrane, except for the working surface where abundant odorous compounds are generated and emitted. The landfill site has often been the subject of complaints regarding olfactory nuisances because some residential communities are located close to the site. The manipulation of fresh garbage over the working surface is the dominant source of odorous compound emissions.

### Gas sampling strategy

Gas sampling was conducted at the landfill in a number of selected days from March 2012 to March 2014, which represented different periods throughout the two years. The sampling points were selected thoughtfully every time since the working surface was shifting according to the manipulation of solid waste disposal. To ensure that these measurements represent actual odor emissions from the working surface of the landfill, the samples were collected at the working surface uncovered with high-density polyethylene membranes. Five or six samples were collected at each sampling spot; three samples were collected at daytime, and two or three at nighttime (Table 1). Meteorological parameters, such as wind speed and direction, atmospheric pressure, ambient temperature, and relative humidity data, were also recorded during sampling in order to evaluate their influences on odorous compound emissions.

**Table 1:** Sampling campaign records.

Sampling date	Sampling time and abbreviation					
	10:00	14:00	18:00	22:00	3:00	6:00
Mar. 27 to 28, 2012	M121	M122	M123	M124	M125	
Aug. 30 to 31, 2012	A121	A122	A123	A124	A125	
Nov. 8 to 9, 2012	N121	N122	N123	N124	N125	
Jan. 23 to 24, 2013	J131	J132	J133	J134	J135	
Mar. 28 to 29, 2013	M131	M132	M133	M134	M135	
Aug. 29 to 30, 2013	A131	A132	A133	A134	A135*	A136
Nov. 25 to 26, 2013	N131	N132	N133	N134	N135*	N136
Mar. 3 to 4, 2014	M141	M142	M143	M144	M145*	M146

*Sample time was at 2:00.

The samples were collected using a specially designed sampler (NO. SOC-01, National Key Laboratory of Odor Pollution Control of Ministry of Environmental Protection, China) at a height of approximately 1.5 m above the working surface of the landfill, which ensured that the samples were obtained at a height similar to that of the respiratory zone of humans. Gas samples were drawn into bi-oriented polyester bags (Environmental Science and Technology Development Co., Ltd, China) using a sampler system, which works based on the lung principle. The internal vacuum pump of the system draws the gas directly into the bag by evacuating the tightly closed atmospheric pressure vessel in which a sampling bag is placed. Odor-free bags that are impermeable to water and organics were used only once. Before sampling, the bag was washed twice by sucking and discharging air at the sampling point to minimize the influence of VOCs that originated from the bag. Polytertrafluoroethylene sampling tubes were used as input and connection lines. Samples were transported to the laboratory and analyzed within 24 h. More details on landfill site descriptions and gas samplings can be found in Duan et al. [19].

### GC—MS analysis

The obtained gas samples were first pre-concentrated using cryogenic liquid nitrogen based on the methodology of EPA TO-14 (US-EPA, 1999a, Compendium Method TO-14). A three-grade cold trap concentrator (Entench 7100, USA) with an injection volume of 400 ml was used. The pre-concentrated gas samples were then passed through a system that consisted of gas chromatography (GC) (Agilent 7890, USA) equipped with a mass selective (MS) detector (Agilent 5975C, USA) for analysis. The GC column was DB-5ms (60 m×0.32 mm×1.0 μm) and was programmed with three different temperature ranges. The temperature range was first kept from 35^°^C to 150^°^C at an increasing rate of 5^°^C min^-1^, then from 150^°^C to 220^°^C at an increasing rate of 15^°^C min^-1^, and then held for 7 min at 220^°^C. The flow rate of the carrier gas (He) was 1.5 mL min^-1^. The detector mass range was set from 15 m/z to 300 m/z, and the ionization voltage was 70 eV. The compounds were identified by comparing their retention times with the said standards, and the identified compounds were quantified using the internal standard method. The detailed GC—MS analysis procedure is described in the study of Duan et al. [19].

### Quality assurance and quality control (QA/QC)

First, all sampling bags that are impermeable to water and organics were used only once. Prior to the sampling, they were analyzed in the laboratory to ensure that no odorous pollutants were present. Before sampling, the bag was washed twice by sucking and discharging the gas at the sampling point. Secondly, the quality assurance and quality control (QA/QC) procedure included blanks, parallel samples and duplicate measurements of samples [4]. Two bags were sampled far away from the landfill during every sampling campaign, which served as blanks. To ensure that there was no contamination from sample collection, transportation or storage during the exposure period, the blanks were transported along with the sampling bags to the sampling points, stored and analyzed in the laboratory. The blanks presented no significant contamination of any odorous compounds. Duplicate samples were also obtained at each sampling points. Concentrations of target odorous compounds in duplicate samples were in good accordance. Finally, when the analysis of VOC is made using sorbent tube, the same material phases between samples and standards should be maintained. As gaseous samples pass through sorbent packed in tube, calibration should also be made identically by passing gaseous standards into tube. Using liquid standards to calibrate gaseous samples may yield a discrepancy, as the liquid phase standard typically has more sensitive response. Thereby, the calibrated results of gas samples may be underestimated [4]. According to Demeestere et al. [24], quantification of gaseous VOCs loaded on a sorbent tube using response factors obtained with liquid standards results in systematic deviations of 40–80%. Considering these reasons, in this study, a new calibration curve was determined by employing gaseous phase standards.

### Data processing method

A MATLAB procedure was applied to estimate the approximate distribution pattern of the data for the different compounds. A MATLAB procedure test was conducted for each compound using the kernel smoothing density estimation (ksdensity) function. This function estimates the probability density value of the samples thereby facilitating the plotting of probability density curves. The approximate distribution pattern of the concentration data for each substance can be determined based on the probability density curve. The arithmetic mean of the substances with normally distributed concentration data was selected as the best estimator of central tendency of such substances. The data for substances without normally distributed concentration data were further checked for log-normal distribution. The geometric mean of these landfill gas constituents was selected as the best estimator of central tendency of such constituents. The average of the arithmetic mean, geometric mean, and median of the data was selected as the default relative concentration for the remaining substances whose data had non-normal or non-log-normal distribution.

## Results and Discussion

### Composition of the odorous compounds

**Fig 1:**
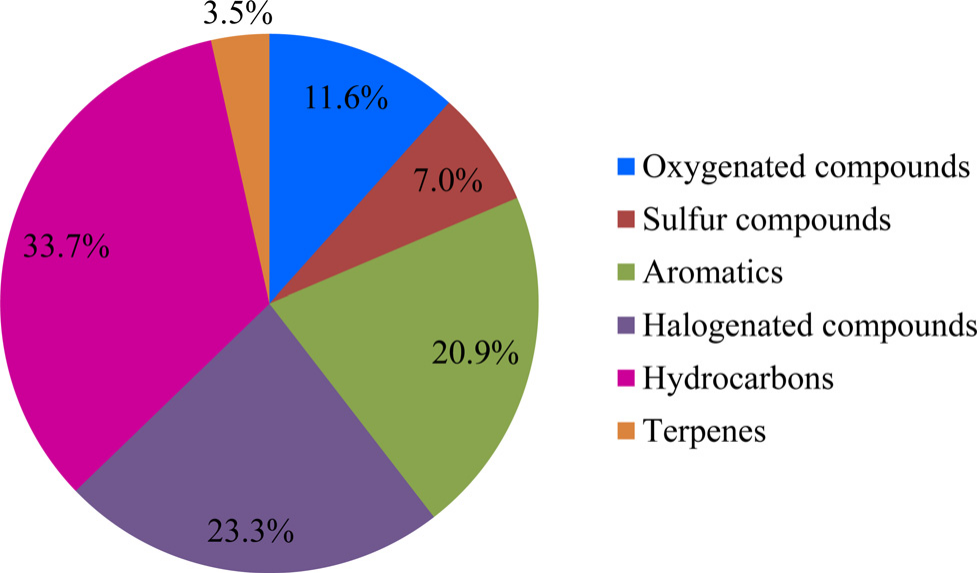
Categories and proportions of the measured odorous compounds from the working surface.

The sampling campaign was conducted at the landfill for eight times on selected days from March 2012 to March 2014, with a total of 43 valid samples obtained. Among these samples, 41, 59, 66, 54, 63, 54, 41, and 42 species of odorous compounds were identified and quantified during the eight sampling activities, respectively, and a number of 86 species of odorous compounds were identified and quantified all together, which demonstrated that the odorous compound emissions indeed varied temporally. The odorous compounds that were measured from the working surface were classified into six categories: oxygenated compounds (alcohols, esters, ethers, ketones, and aldehydes), sulfur compounds, aromatics, halogenated compounds, hydrocarbons (alkanes and alkenes), and terpenes, as shown in Fig. 1.

As it can be seen from Fig. 1, the following compounds were identified and quantified: 10 species of oxygenated compounds (11.6%), 6 species of sulfur compounds (7.0%), 18 species of aromatics (20.9%), 20 species of halogenated compounds (23.3%), 29 species of hydrocarbons (33.7%), and 3 species of terpenes (3.5%). Among the 86 species of odorous compounds detected from the 43 samples, 6 were detected in all 43 samples, including dichloromethane, toluene, ethyl benzene, ethyl alcohol, alpha-pinene, and limonene; 36 were detected in more than 22 samples, with a present frequency higher than 50%; and 60 were detected in more than 9 samples, with a present frequency higher than 20%. The compounds with a present frequency higher than 20% were assigned a high value during the subsequent data processing to ensure that the statistical analysis was scientific.

The total average concentrations of the oxygenated compounds, sulfur compounds, aromatics, halogenated compounds, hydrocarbons, and terpenes were 2.450 mg/m^3^, 0.246 mg/m^3^, 0.203mg/m^3^, 0.319 mg/m^3^, 0.530 mg/m^3^, and 0.217 mg/m^3^, respectively. These findings indicate that oxygenated compounds, halogenated compounds, and hydrocarbons are the main contaminants emitted from the working surface of the landfill. However, the sulfur compounds also serve an important function in causing nuisances related to odor pollution near the landfills because of their low odor threshold.

### Data distribution pattern of different compounds

Estimating the probability density of a sample is necessary when processing data, which means that the corresponding probability distribution curve of a given set of data must be drawn. MATLAB statistical tools directly supply a useful function in the ksdensity function. This function enables the determination of the approximate distribution pattern of the concentration data for each substance. Based on the different distributed types, a different method of calculating the concentration of the substance can then be selected, and the typical concentration of each substance can be obtained. All the 60 species of the compounds whose present frequency was greater than 20% were estimated using the ksdensity function of MATLAB. More details about the method can be found in Section 2. 5.

The approximate distribution patterns of a total of 86 substances were obtained; they are summarized in Table 2. The summary shows that the amount of the 26 kinds of substances detected in the samples was less than 9. To ensure the scientificity of the statistics, the subsequent data processing primarily focused on substances with a detected amount greater than or equal to 9; 60 kinds of substances passed this criterion.

**Table 2:** Distribution patterns of the concentrations of the odorous compounds.

Category	Compound	N/43	Distribution pattern
Oxygenated compounds	Ethyl alcohol	43	log-normal
	Ethyl acetate	33	n-n
	Acetaldehyde	9	log-normal
	Acetone	21	n-n
	2-Butanone	13	n-n
	4-Methyl-2-pentanone	6	TL
	2-Hexanone	5	TL
	Butyraldehyde	1	TL
	Isopropanol	2	TL
	Tert-butyl methyl ether	2	TL
Halogenated compounds	Dichloromethane	43	log-normal
	Chlorobenzene	23	normal
	Difluorodichloromethane	33	normal
	Chloromethane	17	n-n
	Fluoro trichloro methane	42	n-n
	Trichloromethane	28	log-normal
	1,2-Dichloroethane	39	n-n
	Carbon tetrachloride	27	n-n
	1,2-Dichloropropane	21	log-normal
	Perchlorethylene	18	log-normal
	1,4-Dichlorobenzene	32	n-n
	Vinyl chloride	3	TL
	Chloroethane	1	TL
	1,1,2-Trichloro-1,2,2-trifluoroethane	7	TL
	trans-1,2-Dichloroethylene	1	TL
	1,1-Dichloroethane	2	TL
	cis-1,2-Dichloroethylene	7	TL
	Trichloroethylene	6	TL
	1,1,2-Trichloroethane	8	TL
	1,3-Dichlorobenzene	3	TL
Terpenes	alpha-Pinene	43	log-normal
	beta-Pinene	20	log-normal
	Limonene	43	n-n
Sulfur compounds	Carbon disulfide	37	log-normal
	Hydrogen sulfide	20	n-n
	Methanethiol	13	normal
	Dimethyl sulfide	24	n-n
	Ethyl sulfide	10	normal
	Disulfide, dimethyl	25	n-n
Aromatics	Benzene	42	normal
	Toluene	43	log-normal
	Ethylbenzene	43	normal
	Benzene, 1,3-dimethyl-	40	normal
	p-Xylene	34	normal
	Ethenylbenzene	30	log-normal
	o-Xylene	36	normal
	p-Ethyltoluene	29	normal
	Benzene, 1,3,5-trimethyl-	36	log-normal
	Benzene, 1,2,4-trimethyl-	42	log-normal
	Naphthalene	25	log-normal
	Cumene	14	log-normal
	m-Ethyltoluene	23	log-normal
	o-Ethyltoluene	21	n-n
	1,3-Diethylbenzene	13	n-n
	1,4-Diethylbenzene	13	n-n
	n-Propylbenzene	1	TL
	1,2,3-Trimethylbenzene	4	TL
Hydrocarbons	Propylene	37	log-normal
	Hexane	29	n-n
	Cyclohexane	20	n-n
	n-Heptane	26	log-normal
	Propane	35	n-n
	Isobutane	42	n-n
	Butane	42	log-normal
	2-Methylbutane	34	log-normal
	Pentane	29	n-n
	Cyclopentane	21	n-n
	2-Methylpentane	14	log-normal
	3-Methylpentane	16	n-n
	1-Hexene	9	n-n
	Methylcyclopentane	14	log-normal
	Methylcyclohexane	11	n-n
	Octane	13	n-n
	Nonane	10	n-n
	Decane	20	n-n
	Undecane	9	n-n
	Dodecane	6	TL
	1-Butene	1	TL
	cis-2-Butene	7	TL
	1-Pentene	4	TL
	2-Methyl-1,3-butadiene	7	TL
	trans-2-Pentene	2	TL
	2,3-Dimethylbutane	4	TL
	2-Methylhexane	5	TL
	3-Methylhexane	8	TL
	3-Methylheptane	1	TL

Note: N/43: Detected amount among 43 valid samples; n-n: non-normal or non-log-normal distribution; TL: the detected amount is insufficient for analysis (detected amount < 9).

Table 2 shows the following results: Among the 60 kinds of substances whose approximate distribution patterns were determined, 10 approximately matched the normal distribution, 22 approximately matched the log-normal distribution, and the remaining 28 matched neither normal nor log-normal distribution. Of the detected 10 species of oxygenated compounds, 2 (ethyl alcohol and acetaldehyde) approximately matched the normal distribution, and the rest either had insufficient detected amounts for statistics and analysis or did not match the normal or log-normal distribution. Of the detected 20 species of halogenated compounds, 4 (dichloromethane, trichloromethane, 1,2-dichloropropane, and perchlorethylene) approximately matched the log-normal distribution, 2 (chlorobenzene and difluorodichloromethane) approximately matched the normal distribution, and the rest either had insufficient detected amounts for statistics and analysis or did not match the normal or log-normal distribution. Of the 3 detected species of terpenes, alpha-pinene, and beta-pinene approximately matched the normal distribution, whereas limonene matched neither the normal nor log-normal distribution. Of the 6 detected species of sulfur compounds, carbon disulfide approximately matched the log-normal distribution, methyl mercaptan and ethyl sulfide approximately matched the normal distribution, and the other three matched neither the normal nor log-normal distribution. Of the 18 detected species of aromatics, 7 (toluene, ethenylbenzene, 1,3,5-trimethylbenzene, 1,2,4-trimethylbenzene, naphthalene, cumene, and m-ethyltoluene) approximately matched the log-normal distribution, 6 (benzene, ethylbenzene, m-xylene, p-xylene, o-xylene, and p-ethyltoluene) approximately matched the normal distribution, and the rest either had insufficient detected amounts for statistics and analysis or did not match the normal or log-normal distribution. Of the 29 detected species of hydrocarbons, 6 (propylene, n-heptane, butane, 2-methylbutane, 2-methylpentane, and methylcyclopentane) approximately matched the log-normal distribution, none matched the normal distribution, and the others either had insufficient detected amounts for statistics and analysis or did not match the normal or log normal distribution.

### Relative concentrations of ethanol and other odorous compounds

The best estimator for each compound was calculated from the data distribution pattern of the different compounds, as presented in Section 3.2. According to the analysis in the introduction, it is possible and necessary to calculate the best estimator of the concentration for each substance and the relative concentration to ethyl alcohol of each substance. The specific results are summarized in Table 3.

**Table 3:** Concentration and relative concentration of each odorous compound.

Compounds	Geomean mg/m^3^	Arimean mg/m^3^	Median mg/m^3^	Best estimator mg/m^3^	ppm	Relative ppm
Ethyl alcohol	0.6803	1.2806	0.6998	0.6803	0.3308	1000.00
Ethyl acetate	0.0509	0.0882	0.0433	0.0608	0.0154	46.70
Acetaldehyde	0.2170	0.4655	0.3800	0.2170	0.1103	333.57
Acetone	0.0413	0.0615	0.0385	0.0471	0.0182	54.91
2-Butanone	0.0377	0.0656	0.0649	0.0561	0.0174	52.66
Dichloromethane	0.0166	0.0295	0.0161	0.0166	0.0044	13.26
Chlorobenzene	0.0019	0.0036	0.0025	0.0036	0.0007	2.18
Difluorodichloromethane	0.0038	0.0101	0.0072	0.0101	0.0012	3.61
Chloromethane	0.0024	0.0050	0.0012	0.0028	0.0013	3.82
Fluoro trichloro methane	0.0298	0.1121	0.0343	0.0587	0.0096	28.95
Trichloromethane	0.0015	0.0028	0.0022	0.0015	0.0002	0.67
1,2-Dichloroethane	0.0113	0.0180	0.0098	0.0130	0.0029	8.89
Carbon tetrachloride	0.0005	0.0010	0.0006	0.0007	0.0001	0.31
1,2-Dichloropropane	0.0124	0.0195	0.0130	0.0124	0.0025	7.45
Perchlorethylene	0.0065	0.0138	0.0065	0.0065	0.0009	2.66
1,4-Dichlorobenzene	0.0066	0.0112	0.0055	0.0078	0.0012	3.57
alpha-Pinene	0.0175	0.0207	0.0154	0.0175	0.0029	8.71
beta-Pinene	0.0193	0.0230	0.0160	0.0193	0.0032	9.61
Limonene	0.0833	0.1732	0.0922	0.1162	0.0153	46.22
Carbon disulfide	0.0057	0.0133	0.0075	0.0057	0.0017	5.08
Hydrogen sulfide	0.0381	0.0459	0.0349	0.0396	0.0260	78.73
Methanethiol	0.0118	0.0228	0.0159	0.0228	0.0106	32.07
Dimethyl sulfide	0.0139	0.0201	0.0144	0.0161	0.0058	17.57
Ethyl sulfide	0.0245	0.0304	0.0296	0.0304	0.0075	22.82
Disulfide, dimethyl	0.0530	0.0727	0.0430	0.0563	0.0134	40.44
Benzene	0.0114	0.0154	0.0103	0.0154	0.0044	13.35
Toluene	0.0229	0.0351	0.0219	0.0229	0.0056	16.79
Ethylbenzene	0.0142	0.0249	0.0153	0.0249	0.0053	15.88
Benzene, 1,3-dimethyl-	0.0116	0.0207	0.0135	0.0207	0.0044	13.23
p-Xylene	0.0082	0.0148	0.0082	0.0148	0.0031	9.44
Ethenylbenzene	0.0059	0.0078	0.0061	0.0059	0.0013	3.81
o-Xylene	0.0087	0.0145	0.0078	0.0145	0.0031	9.22
p-Ethyltoluene	0.0030	0.0051	0.0040	0.0051	0.0009	2.86
Benzene, 1,3,5-trimethyl-	0.0018	0.0035	0.0019	0.0018	0.0003	1.02
Benzene, 1,2,4-trimethyl-	0.0080	0.0142	0.0079	0.0080	0.0015	4.52
Naphthalene	0.0101	0.0116	0.0109	0.0101	0.0018	5.33
Cumene	0.0004	0.0005	0.0005	0.0004	0.0001	0.24
m-Ethyltoluene	0.0038	0.0064	0.0029	0.0038	0.0007	2.12
o-Ethyltoluene	0.0022	0.0032	0.0018	0.0024	0.0004	1.34
1,3-Diethylbenzene	0.0022	0.0025	0.0018	0.0021	0.0004	1.08
1,4-Diethylbenzene	0.0027	0.0039	0.0021	0.0029	0.0005	1.47
Propylene	0.0243	0.0427	0.0255	0.0243	0.0130	39.15
Hexane	0.0060	0.0088	0.0095	0.0081	0.0021	6.35
Cyclohexane	0.0045	0.0088	0.0050	0.0061	0.0016	4.90
n-Heptane	0.0055	0.0106	0.0061	0.0055	0.0012	3.71
Propane	0.0318	0.0538	0.0277	0.0378	0.0192	57.98
Isobutane	0.0439	0.0939	0.0398	0.0592	0.0228	68.94
Butane	0.0386	0.0782	0.0386	0.0386	0.0149	44.96
2-Methylbutane	0.0175	0.0240	0.0173	0.0175	0.0054	16.43
Pentane	0.0167	0.0288	0.0143	0.0199	0.0062	18.70
Cyclopentane	0.0038	0.0081	0.0034	0.0051	0.0016	4.91
2-Methylpentane	0.0029	0.0034	0.0031	0.0029	0.0008	2.27
3-Methylpentane	0.0036	0.0067	0.0030	0.0044	0.0012	3.50
1-Hexene	0.0017	0.0020	0.0016	0.0018	0.0005	1.41
Methylcyclopentane	0.0029	0.0091	0.0055	0.0029	0.0008	2.31
Methylcyclohexane	0.0014	0.0041	0.0020	0.0025	0.0006	1.72
Octane	0.0046	0.0100	0.0028	0.0058	0.0011	3.42
Nonane	0.0087	0.0439	0.0190	0.0239	0.0042	12.61
Decane	0.0069	0.0232	0.0050	0.0117	0.0018	5.55
Undecane	0.0023	0.0162	0.0069	0.0085	0.0012	3.67

Table 3 shows the approximate distribution pattern, geometric mean concentration (Geomean), arithmetic mean concentration (Arimean), median concentration (Median), and volume concentration (in ppm) of each substance. It also provides the concentrations of various substances with respect to the concentration of ethyl alcohol (1000 ppm) in the last column. In other words, identifying the actual concentration of ethyl alcohol facilitates calculating the concentration of the remaining compounds based on the above results, which could make significance to the research and control of odorous compounds emitted from landfills.

### Comparison of the relative concentration for LandGEM and MoLandge

A comparison of the relative concentration of the results obtained from the current study (i.e., referred to as Model of Landfill gas emission (MoLandge), developed by our laboratory) and those from LandGEM (EPA, 2005) are summarized in Table 4.

**Table 4:** Comparison of the relative concentration of LandGEM and MoLandge (after uniformization).

	LandGEM	MoLandge	LandGEM	MoLandge
	(relative ppm)		(after uniformization)	
Hydrogen sulfide	36	78.73	1000	1000
1,2-Dichloroethane	0.41	8.89	11.39	112.92
1,2-Dichloropropane	0.18	7.45	5.00	94.63
Acetone	7	54.91	194.44	697.45
Benzene	11	13.35	305.56	169.57
Butane	5	44.96	138.89	571.07
Carbon disulfide	0.58	5.08	16.11	64.52
Carbon tetrachloride	0.004	0.31	0.11	3.94
Chlorobenzene	0.25	2.18	6.94	27.69
Chloroform	0.03	0.67	0.83	8.51
Chloromethane	1.2	3.82	33.33	48.52
Dichlorobenzene	0.21	3.57	5.83	45.34
Dichlorodifluoromethane	16	7.75	444.44	98.44
Dichloromethane	14	13.26	388.89	168.42
Dimethyl sulfide	7.8	17.57	216.67	223.17
Ethylbenzene	4.6	15.88	127.78	201.70
Fluorotrichloromethane	0.76	28.95	21.11	367.71
Hexane	6.6	6.35	183.33	80.66
Methyl mercaptan	2.5	32.07	69.44	407.34
Pentane	11	18.7	305.56	237.52
Perchloroethylene	3.7	2.66	102.78	33.79
Propane	11	57.98	305.56	736.44
Xylenes	12	31.89	333.33	405.06

LandGEM lists the relative concentrations of 46 species of odorous compounds and the current study lists the relative concentrations of 60 species. The two lists have 23 species in common: 4 species of sulfur compounds (hydrogen sulfide, carbon disulfide, dimethyl sulfide, and methyl mercaptan), 1 species of oxygenated compound (acetone), 3 species of aromatics (benzene, ethyl benzene, and xylenes), 4 species of hydrocarbons (butane, hexane, pentane, and propane), and 11 species of halogenated compounds (1,2-dichloroethane, 1,2-dichloropropane, carbon tetrachloride, chlorobenzene, chloroform, chloromethane, dichlorobenzene, dichlorodifluoromethane, dichloromethane, fluorotrichloromethane, and perchloroethylene). The abundant halogenated compounds found in common could have originated from the waste components, some of which are the chief sources of these compounds. These sources include soap, paint, paint remover, industrial solvents, foam blowing agents, and varnish refrigerants, which are all found in the landfill waste. In addition, the emission of halogenated compounds is not influenced completely by biological degradation processes [19].

**Fig 2:**
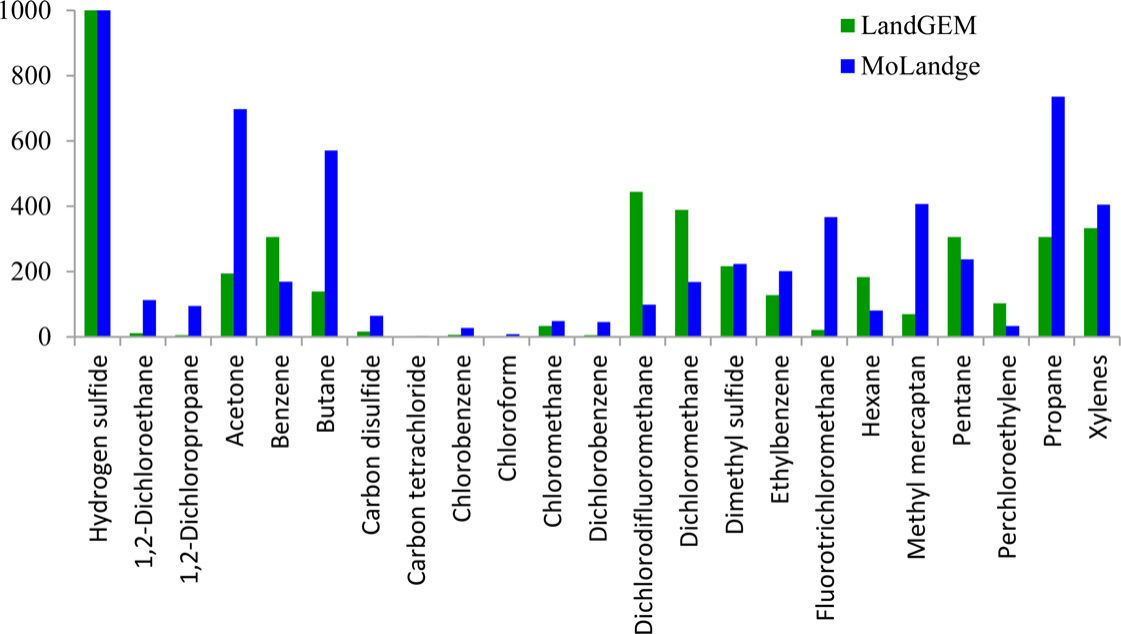
Comparison of the relative concentrations of LandGEM and MoLandge (after uniformization).

Hydrogen sulfide was selected as the normalized index compound because it exhibited a similar order of magnitude in the results of both LandGEM and MoLandge, where its relative ppm is 36 in LandGEM and 78.73 in MoLandge. Taking hydrogen sulfide as 1000 (i.e., after normalization, dimensionless), various compounds from both LandGEM and MoLandge were compared; the results are illustrated in Fig. 2.

As shown in Fig. 2, most of the odorous compounds in the two data series shared similar magnitudes. This result indicates that the landfill gas emitted from the US and China generally contains similar constituents. The proportion of most compounds (17 out of 23) in MoLandge is higher than that in LandGEM, except for benzene, dichlorodifluoromethane, dichloromethane, hexane, pentane, and perchloroethylene. As to the 17 species (i.e., high proportions in MoLandge), the proportion of 6 compounds in MoLandge (1,2-dichloroethane, 1,2-dichloropropane, carbon tetrachloride, chloroform, fluorotrichloromethane, and methyl mercaptan) is higher by one order of magnitude or more than that in LandGEM. The proportion of the remaining 11 compounds in both data series generally has the same order of magnitude.

### Top ten contaminants emitted from landfill

Simultaneously, the contaminants with the maximum concentration were also considered statistically. The top ten contaminants in terms of mass concentration were ethyl alcohol, acetaldehyde, limonene, ethyl acetate, isobutane, trichlorofluoromethane, dimethyl disulfide, 2-butanone, acetone, and hydrogen sulfide, with mass concentrations ranging from 0.0396 mg/m^3^ to 0.6803 mg/m^3^. The top ten contaminants in terms of volume concentration were ethyl alcohol, acetaldehyde, hydrogen sulfide, isobutane, propane, acetone, 2-butanone, ethyl acetate, limonene, and butane, with volume concentrations ranging from 0.0149 ppm to 0.3308 ppm. This study also surveyed and investigated the research findings from the Ministry of the Environment of Japan (JP MOE) and those from the United States Environmental Protection Agency (US EPA) [25], [26]; this step was undertaken to further assess the situation of odor pollution around landfills and therefore obtain the olfactory threshold of most of the substances detected in the present study. Table 5 provides a summary list of the top ten contaminants (characterized in dilution multiples) from the working surface of the studied landfill, along with their concentrations, olfactory thresholds, and dilution multiples. The greater the dilution multiple, the greater the contribution of that species of contaminant made to odor pollution.

**Table 5:** Top ten contaminants (characterized in dilution multiples).

Compounds	Mass concentration (mg/m^3^)	Volume concentration (ppm)	Odor threshold (ppm)	Dilution ratio
Ethyl sulfide	0.0304	0.0075	0.000033	228.709
Methyl mercaptan	0.0228	0.0106	0.00007	151.530
Acetaldehyde	0.2170	0.1103	0.0015	73.562
Hydrogen sulfide	0.0396	0.0260	0.00041	63.522
Disulfide, dimethyl	0.0563	0.0134	0.0022	6.080
Dimethyl sulfide	0.0161	0.0058	0.003	1.937
1,4-Diethylbenzene	0.0029	0.0005	0.00039	1.248
Ethyl alcohol	0.6803	0.3308	0.52	0.636
Limonene	0.1162	0.0153	0.038	0.402
alpha-Pinene	0.0175	0.0029	0.018	0.160

According to the discussions above, the dominant contaminants that cause odor pollution around the landfill are ethyl sulfide, methyl mercaptan, acetaldehyde, and hydrogen sulfide, whose dilution multiples are higher than 60; dimethyl disulfide and dimethyl sulfide also make a certain contribution. Although ethyl alcohol, limonene, and some other substances have higher concentrations, they do not significantly contribute to odor pollution because they have higher odor threshold values.

## Conclusions

This paper presented a thorough and systematic study on the odorous compounds emitted from the landfill working surface and attempted to find the correlations among the odorous compounds using statistical tools. Two main conclusions can be drawn.

First, odorous compound emissions from the working surface were investigated and a significant amount of odorous compounds under six different categories (oxygenated compounds, halogenated compounds, terpenes, sulfur compounds, aromatics, and hydrocarbons) were identified and quantified; their total concentrations ranged from 0.217 mg/m^3^ to 2.450 mg/m^3^. The oxygenated compounds, halogenated compounds, and hydrocarbons were the most abundant contaminants emitted from the landfill working surface and ethyl alcohol presented a highest concentration all over the odorous compounds detected.

Secondly, ethyl alcohol was employed as a new index to estimate the emission of other compounds and the relative concentration of the odorous compounds with respect to the concentration of ethyl alcohol (in 1000 ppm) was determined. Along with the approximate distribution patterns, geometric mean concentrations (Geomean), arithmetic mean concentrations (Arimean), median concentrations (median), and volume concentrations (in ppm) of each substance were determined. These results could facilitate estimating the approximate concentration of other odorous compounds, depending on the concentration of ethyl alcohol.
